# A Study on the psychometric properties of the Chinese version of the Parent Tic Questionnaire

**DOI:** 10.1371/journal.pone.0307948

**Published:** 2025-02-11

**Authors:** Qiang Ding, Douglas W. Woods, Kathryn E. Barber, Wen Xu, Ying Zhao, Shuqin Shen, Jinhua Sun

**Affiliations:** 1 Department of Psychological Medicine, Children’s Hospital of Fudan University, National Children’s Medical Center, Shanghai, China; 2 Department of Psychology, Marquette University, Milwaukee, WI, United States of America; BSMHFT National Centre for Mental Health, UNITED KINGDOM OF GREAT BRITAIN AND NORTHERN IRELAND

## Abstract

**Introduction:**

The Parent Tic Questionnaire (PTQ) is a tool for parents to assess their child’s tic severity, but its effectiveness in non-Western contexts like China has not been thoroughly examined. This study aimed to evaluate the psychometric properties of the PTQ in assessing motor and vocal tic severity among Chinese children diagnosed with tic disorders.

**Method:**

Parents of 268 Chinese children and adolescents aged 6–17 years, diagnosed with tic disorders, completed the PTQ. The study assessed tic severity using the Yale Global Tic Severity Scale (YGTSS) and PTQ. Additionally, obsessive-compulsive symptoms were measured using the Children Yale-Brown Obsessive Compulsive Scale (CY-BOCS), and other behavioral problems were evaluated using the Conners’ Parent Rating Scale (CPRS-48).

**Results:**

The PTQ showed acceptable to good internal consistency (Cronbach’s alpha = 0.67 for motor, 0.77 for vocal, and 0.79 for total tic scores) and acceptable to good two-week test-retest reliability (intraclass correlations, ICC = 0.74 for motor, 0.81 for vocal, and 0.79 for total tic scores). It demonstrated good convergent validity with the YGTSS and effective discriminant validity from obsessive-compulsive and internalizing symptoms (i.e., anxiety and psychosomatic). Furthermore, our analysis revealed significant variability in item difficulty across the questionnaire, indicating differences in how various tics are perceived and reported by Chinese parents, which may influence the assessment’s accuracy and reliability.

**Discussion:**

The findings indicate that the Chinese version of the PTQ is a reliable and valid tool for assessing tic severity in Chinese children with tic disorders, offering significant implications for clinical assessment in diverse cultural contexts. Additionally, our findings on item difficulty highlight the need for further cultural adaptations of the PTQ.

## Introduction

Tic disorders are neurodevelopmental disorders marked by sudden, rapid, repetitive movements and/or vocalizations. Tics can be brief and temporary (such as in Provisional Tic Disorder PTD), but can also persist for longer than a year, leading to diagnoses of Tourette syndrome (TS) or Persistent Motor or Vocal Tic Disorder (PMVT) [1]. In mainland China, tic disorders have a prevalence rate of 2.5% [2]. Often, these disorders coexist with conditions like obsessive-compulsive disorder (OCD) and attention-deficit/hyperactivity disorder (ADHD) [3].

Tics can wax and wane over time, and the presentation of tic disorders is variable. The Yale Global Tic Severity Scale (YGTSS) [4] has been the gold-standard measure of tic severity and is widely used in clinical and research environments in China [5]. The YGTSS is a semi-structured clinician-administered instrument that can take between 30 to 45 minutes to complete. Due to its time-intensive nature and the necessity for trained administrators, the YGTSS is not often used to track regular clinical progresss [6]. This aligns with the broader trend of preferring quicker measures over more time-consuming clinician-rated scales [7]. The YGTSS is also somewhat limited in that it assesses all motor and vocal tics across subdomains, rather than gathering information about individual tics.

Parent- and self-report tic severity scales can provide a convenient alternative to the YGTSS, enabling quick completion in waiting areas prior to treatment sessions. Notably, the Parent Tic Questionnaire (PTQ) [6] distinguishes itself due to its ease of administration. Designed to assess the severity of tics over the preceding week and following the format of the YGTSS [6], the PTQ asks parents to identify the presence or absence of 14 common vocal and 14 common motor tics and subsequently rate the frequency and intensity of each endorsed tic. Frequency is rated (1–4) as weekly, daily, hourly, or constantly, while intensity is rated on a scale of 1 to 4, with higher values denoting more intense tics. The PTQ produces severity scores for both motor and vocal tics by summing frequency and intensity ratings for tics in each category. Motor and vocal tic scores are then combined to yield a total tic severity score.

Preliminary data provided support for the PTQ as both a reliable and accurate tool for gauging tic severity in children [6]. Another study examined the psychometric properties of the PTQ and benchmark scores for determining treatment response [8]. The sample included 126 children aged 9–17 who participated in a randomized control trial of Comprehensive Behavioral Intervention for Tics (CBIT). In this sample, the PTQ was found to be internally consistent (α = 0.80 to 0.86) and temporally stable (ICC = 0.84 to 0.89). It also showed strong validity when compared with YGTSS and could differentiate tic severity from measures of other symptoms like hyperactivity and obsessive-compulsive behavior. A reduction of 55% or a 10-point decrease in PTQ score was deemed a positive treatment response. This study supported the PTQ as a reliable tool for clinicians to gauge meaningful changes in tic severity during treatment [8].

A recent study investigated the psychometric properties of the PTQ across different countries [9]. Drawing from parent-reported data of 223 children diagnosed with Tourette’s disorder (including TS and PMVD) in the United States, the United Kingdom, and a combined cohort from the Netherlands and Norway, Stiede et al [9] explored into cross-cultural patterns concerning tic severity and reactions. The psychometric findings for the PTQ supported its stability and flexibility in evaluating tic severity within these diverse regions.

Previous studies have validated the PTQ in Western contexts, but its effectiveness in non-Western settings, particularly in China, remains underexplored. Given China’s distinct cultural and linguistic characteristics, this study aims to assess both the reliability and validity of the PTQ’s Chinese version and to identify item-level challenges. By analyzing item difficulty, we seek to adapt the PTQ for greater cultural relevance in diagnosing tic disorders among Chinese children.

## Methods

The study was conducted in two phases: 1) the adaptation and translation of the Parent Tic Questionnaire (PTQ) into Chinese for children, and 2) the assessment of the psychometric properties of the Chinese version of the PTQ.

### Phase 1—Translation and cross-cultural adaptation of the PTQ

The PTQ was initially translated into Chinese by a child psychiatrist and two psychological assessors at the Department of Psychological Medicine at Children’s Hospital of Fudan University. This translation was then back-translated into English by two psychological assessors fluent in English. The back-translation was reviewed and compared with the original English version by one of the original authors of the PTQ, D.W. Woods. Based on the feedback, the Chinese version was edited and finalized.

### Phase 2—Assessment of the Psychometric properties

The ethics committees of Children’s Hospital of Fudan University approved this study (NO:2021–386). All parents and children read and signed appropriate consent and assent forms prior to participation.

To gather data, a suite of questionnaires, including the newly adapted PTQ, was administered to participants during routine outpatient visits. A key aspect of this phase was the evaluation of the test-retest reliability of the Chinese PTQ. For this purpose, a random subset of 31 participants was selected from the initial sample of 268. This selection was made to ensure a focused and efficient evaluation of the questionnaire’s stability over time.

#### Participants

This study involved 280 children and adolescents diagnosed with tic disorders (including provisional tic disorder, persistent motor/phonic tic disorder, and Tourette syndrome) according to the DSM-5 criteria. All participants in this study were Chinese. Twelve participants who did not complete the PTQ or other scales were excluded, leaving a final sample of 268 patients (213 male; *M* age = 9.32, SD = 2.78, range = 6–17). Among the respondents, 193 were mothers, 61 were fathers, and 14 were other relatives, including grandparents and aunts (refer to Table 1 for detailed demographics). All participants were recruited from the Department of Psychological Medicine at Children’s Hospital of Fudan University in Shanghai between October 1st, 2021, and December 31st, 2022.

**Table 1:** Demographic profile of respondents of the PTQ.

	Number	Percentage
**Respondent**		
Father	61	22.76%
Mother	193	72.01%
Others (including grandparents or aunt)	14	5.23%
**Education Level of Father**		
Less than High School	47	17.54%
High school or technical secondary school	39	14.55%
Junior College	63	23.51%
Undergraduate degree	89	33.21%
Postgraduate degree and above	30	11.19%
**Education Level of Mother**		
Less than High School	39	14.55%
High school or technical secondary school	46	17.16%
Junior College	60	22.39%
Undergraduate degree	105	39.18%
Postgraduate degree and above	18	6.72%

#### Study variables and instruments

The Chinese version of the PTQ [6], Yale Global Tic Severity Scale (YGTSS) [4], Children’s Yale-Brown Obsessive Compulsive Scale (CY-BOCS) [10], Conners’ Parent Rating Scale (CPRS-48) [11] were administered.

As described before, the PTQ is a parent-report measure used to assess the severity of their child’s tics. The PTQ measures the frequency and intensity of each tic and provides severity scores for motor tics, vocal tics, and a combined total tic score. The motor tic and vocal tic scores each can go up to 112, and the total score can range from 0 to 224 [6].

The YGTSS is a semi-structured clinician-administered interview designed to evaluate the severity of motor and vocal tics over the previous week. The interview assesses the number, frequency, intensity, complexity, and interference of motor and vocal tics. The YGTSS yields total motor and phonic tic scores (ranging from 0–25) that are combined to produce a total tic score (ranging from 0–50). The YGTSS also has a tic impairment rating scored from 0–50 [4]. For the purposes of the present investigation, only the total tic score was included in the analyses.

The CY-BOCS is a modified version of the Yale-Brown Obsessive-Compulsive Scale (Y-BOCS) tailored for use in children. It is a clinician-rated, semi-structured tool that evaluates the severity of obsessions and compulsions. The CY-BOCS includes five items assessing obsessive symptoms and five assessing compulsive symptoms, each scored on a five-point ordinal scale ranging from 0 (none) to 4 (extreme). The total severity score is calculated by summing the scores of all items, with a range of 0 to 40. The instrument has been shown to be reliable and valid for use in children with OCD with substantial evidence from various countries [10].

The CPRS-48 is a parent-report scale commonly used to assess ADHD symptoms and related emotional and behavioral issues in children. The CPRS-48 scale is comprised of 48 questions. In the Chinese version of CPRS-48, the homogeneity reliability of Cronbach α was 0.93, the correlation of Spearman-brown split-half was 0.90, and the retest reliability of the total score was 0.59 [11]. Parents answer each question using a 4-point scale ranging from 0 (“not at all true”) to 3 (“very much true”), with a higher score indicating more severe behavioral problems. The revised version of the CPRS-48 employs six subscales to evaluate various behavioral outcomes: conduct problems, learning problems, psychosomatic problems, impulsive-hyperactive, anxiety, and ADHD [11].

### Data analysis

Statistical analysis was performed using SPSS software (Version 24.0, SPSS Inc., Chicago, IL, USA).

First, we evaluated the relevance of each item on the PTQ by calculating item-total correlations. This analysis helped determine how well each item correlates with the overall construct it measures. Concurrently, we computed item difficulty for each item, which reflects how frequently respondents endorsed each item. Item difficulty is quantified by the proportion of participants reporting the presence of a tic; higher values indicate that more participants reported the tic, suggesting a lower item difficulty.

To assess the validity and reliability of the Chinese version of the PTQ, several statistical analyses were conducted. We analyzed the internal consistency of the PTQ by calculating Cronbach’s alpha for each subscale. Additionally, to explore the impact of individual items on the scale’s reliability, we recalculated Cronbach’s alpha after removing each item. Items that increase the alpha when removed may be detracting from the scale’s internal consistency. For this dataset, α values ≥ 0.90 were considered excellent, 0.70 to 0.89 good, 0.60 to 0.69 acceptable, and < 0.60 poor [12]. Test-retest reliability was determined using one way random effects ICCs based on data from 31 participants who completed the retest after two weeks(time frame:14–18 days). ICC of 0.80 to 1.00 was indicative of excellent test-retest reliability, values of 0.60 to 0.79 signified acceptable reliability [13]. Correlations were calculated between PTQ subscales and total scores and YGTSS subscales and total scores to test concurrent validity. A correlation value of >0.50 between the PTQ and YGTSS indicated good concurrent validity. Correlations of 0.30 to 0.49 and 0.10 to 0.29 represented fair and poor concurrent validity, respectively [14]. Discriminant validity was evaluated by conducting correlations between PTQ total scores and scores on the CY-BOCS and CPRS-48 subscales. Good discriminant validity was represented by correlations of 0.10 to 0.29 between the PTQ and CY-BOCS, CPRS-48 subscales. Correlation values that exceeded this range were considered fair (0.30 to 0.49) and poor (> 0.50) discriminant validity [14].

## Results

### Results of evaluation with the PTQ and other measures

The mean PTQ total score was 26.57±20.43 points. The mean YGTSS tic severity score was 22.26±9.2 points. The mean scores of the CY-BOCS and CPRS-48 sub-scales are reported in Table 2.

**Table 2:** Summary of clinical assessment.

	Mean	SD
**PTQ**		
Motor tic subscale	14.24	11.51
Vocal tic subscale	12.33	13.48
Total score	26.57	20.43
**YGTSS**		
Motor tic subscale	12.40	5.48
Vocal tic subscale	9.69	7.18
Tic severity score	22.09	9.24
**CYBOCS**		
Obsessions subscale	9.16	3.79
Compulsions subscale	8.52	3.38
Total score	17.68	6.71
**CPRS-48**		
Conduct problems subscale	0.77	0.53
Learning problems subscale	1.23	0.77
Psychosomatic problems subscale	0.22	0.30
Impulsive-hyperactive behaviors	1.06	0.74
Anxiety subscale	0.59	0.53
ADHD index	0.97	0.60

### Item-total correlations and item difficulty analysis of the PTQ

The item-total correlations were significant across all items, indicating a strong relationship between individual items and the overall subscale scores. Item difficulty across both the motor and vocal tic subscales showed variability, indicating differences in how frequently certain tics are reported by respondents. For the motor tic subscale: difficulty levels ranged from low (Item11 at 0.09, Item12 at 0.08 and Item 8 at 0.05) to moderate (Item 1 at 0.57 and Item 3 at 0.46); vocal tic items also displayed a broad range of difficulty, with Item 1, 4, 14 exhibiting moderate difficulty (range from 0.4 to 0.54), indicating a common occurrence, whereas Items 6 to item 9, and Item 12, 13 showed lower difficulties (range from 0.06 to 0.09), suggesting these are less frequently reported(see Table 3 for details).

**Table 3:** Item-total correlations, item difficulty, and changes in total score cronbach’s alpha if item removed for PTQ items.

PTQ_Motor tic subscale	Item 1	Item 2	Item 3	Item 4	Item 5	Item 6	Item 7
PTQ_Total score	.21***	.26***	.31***	.26***	.27***	.42***	.48***
Item difficulty	.57	.31	.46	.13	.26	.37	.17
α if item removed	.79	.79	.79	.78	.79	.78	.77
**PTQ_Motor tic subscale**	Item 8	Item 9	Item 10	Item 11	Item 12	Item 13	Item 14
PTQ_Total score	.40***	.41***	.43***	.40***	.41***	.30***	.52***
Item difficulty	.05	.24	.31	.09	.08	.12	.24
α if item removed	.78	.78	.78	.78	.78	.78	.77
**PTQ_Vocal tic** **subscale**	Item 1	Item 2	Item 3	Item 4	Item 5	Item 6	Item 7
PTQ_Total_Score	.59***	.38***	.42***	.47***	.37***	.46***	.41***
Item difficulty	.4	.31	.13	.54	.1	.09	.08
α if item removed	.77	.78	.78	.78	.78	.78	.78
**PTQ_Vocal tic** **subscale**	Item 8	Item 9	Item 10	Item 11	Item 12	Item 13	item 14
PTQ_Total_Score	.37***	.43***	.44***	.37***	.33***	.63***	.60***
Item difficulty	.09	.09	.19	.12	.06	.09	.40
α if item removed	.78	.78	.78	.78	.78	.77	.77

*p< 0.05

**p<0.01

***p<0.001.

### Reliability verification

**Internal consistency.** Cronbach’s alpha coefficients indicated that the PTQ motor tic scale had acceptable internal consistency(α = 0.67). The vocal tic scale (α = 0.77) and total score (α = 0.79) showed acceptable to good internal consistency. Changes in Cronbach’s alpha upon the removal of items varied slightly, α values were fairly consistent upon the removal of items, often remaining unchanged, which supports the internal consistency of the total score.

### Test-retest reliability

Two-week test-retest reliability of the PTQ Motor tic score (ICC = 0.74; 95% CI = 0.53 to 0.86), PTQ Vocal tic score (ICC = 0.81; 95% CI = 0.64 to 0.90), and PTQ Total tic score (ICC = 0.79; 95% CI = 0.61 to 0.89) were acceptable to good (n = 31).

### Concurrent validity

Correlations between YGTSS and PTQ scores were strong for motor tic severity (r = 0.67, *p <* .001), vocal tic severity (r = 0.71, *p <* .001), and total tic severity score (r = 0.68, *p <* .001).

### Discriminant validity

Total PTQ score was not significantly correlated with the CY-BOCS obsessions score (r = 0.11, *p =* .073), compulsions score (r = 0.002, *p =* .970), or total score (r = 0.063, *p =* .303). PTQ scores were also not significantly correlated with the CPRS-48 subscales of conduct problems (r = 0.11, *p =* .072), anxiety (r = 0.009, *p =* .884), or psychosomatic problems (r = 0.006, *p =* .921). PTQ showed significant small correlations with the CPRS-48 impulsive behaviors (r = 0.16, *p =* .009), study problems (r = 0.14, *p =* .022), and hyperactive behaviors (r = 0.15, *p =* .014) subscales.

### Inter-scale correlations

Inter-scale correlations between the PTQ total tic score and PTQ motor tic (r = 0.78, *p* < .001) and vocal tic (r = 0.85, *p*< .001) scores were strong. The correlation between PTQ motor tic and vocal tic scores was fair (r = 0.33, *p*< .001).

## Discussion

This study is the first to investigate the psychometric properties of the PTQ in Chinese children with tic disorders. Our research introduces an additional tool for tic disorder assessment in China, as a complement to the YGTSS. As a parent-report measure, the PTQ offers an efficient way to regularly measure tic severity, saving time for clinical evaluators in China.

### Item difficulty

The analysis of the PTQ’s item difficulties offers substantial insights into the reporting patterns of tic symptoms among Chinese children. Items including eye blinking (motor tic Item 1), head jerking (motor tic Item 3), chest/stomach tightening (motor tic Item 7), grunting (vocal tic Item 1), coughing (vocal tic Item 4), and complex vocal combinations (vocal tic Item 14) exhibited higher difficulty scores, indicating they are more frequently observed and reported. The visibility and potential social disruptiveness of these symptoms likely make them more recognizable to parents, who may prioritize these as significant concerns warranting medical attention. Such symptoms are typically more disruptive in social settings, thereby drawing more attention and increasing the likelihood of reporting, consistent with findings from broader studies on tic disorders [15,16].

Conversely, symptoms such as pelvic tensing movements (motor tic Item 8), echopraxia (motor tic Item 11), copropraxia (motor tic Item 12), syllables (vocal tic Item 6), echolalia (vocal tic Item 9), and other vocal tics (vocal tic Items 12 and 13), despite their clinical significance, had lower difficulty scores. These findings suggest underreporting, which could stem from cultural reticence or misconceptions about their neurological basis, affecting symptom recognition and reporting practices [17,18].

To further refine the PTQ for use in China, adaptations could include more detailed descriptions and culturally specific examples of less commonly reported tics. Such modifications might help parents better understand and identify the symptoms of tic disorders, leading to more accurate and comprehensive reporting.

### Reliability of the PTQ

Our research demonstrated acceptable internal consistency for the PTQ. The item-total correlations were significant across all items, indicating a robust relationship between individual items and the overall subscale scores. This finding underscores the internal coherence of the PTQ, demonstrating that each item contributes effectively to the composite measure of tic severity.

However, the PTQ showed slightly lower internal consistency in this sample as compared to previous Western-based studies (0.68–0.79 in our study, vs. 0.77–0.92 in studies by Western-based studies) [6,8,9]. Such variations may be attributable to profound cultural and contextual factors. In different cultures, the perception and interpretation of symptoms, such as motor or vocal tics, might differ [19]. Noticeable tics in a Western context might be seen as harmless or habitual in the Chinese cultural environment. For example, Western parents may identify a child’s Copropraxia or syllables as motor or vocal tics, respectively, while Chinese parents might dismiss these behaviors as temporary childhood habits or responses to their surroundings. This cultural perspective, combined with the stigma attached to neurological or psychiatric conditions (e.g., some Chinese parents may fear negative judgments, therefore minimizing or omitting reports of certain behaviors when completing the PTQ) may influence how tics manifest or are reported [17,20]. Additionally, cultural perceptions of what constitutes "severity" could vary, impacting the consistency of responses on severity-related items of the PTQ [9]. Parallel to the study by Ricketts et al. [8], our research found a comparable mean YGTSS severity score (22.09 in this study vs. 24.66 by Ricketts et al.[8]). Yet, the PTQ total scores were notably lower in our sample (26.57) compared to Ricketts et al.’s (36.11) [8], suggesting that Chinese parents might perceive less severity than their U.S counterparts. Given these complex cultural subtleties, our results emphasize the importance of culturally-tailored adaptations or guidelines when employing tools like the PTQ in diverse settings, such as China. Moreover, in previous studies [6,8], the participants included only those with PMVT and TS, while our study also incorporates individuals with PTD, which may potentially impacts the internal consistency of the PTQ. Notably, our analysis of changes in Cronbach’s alpha with the removal of individual items did not indicate substantial improvements in internal consistency, suggesting that no single item disproportionately affects the overall scale reliability.

The two-week test-retest reliability for the PTQ was 0.74 for motor tics, 0.81 for vocal tics, and 0.79 for total tic severity in our study. Compared to the findings of Chang et al.[6] and Ricketts et al.[8], the reliability in a Chinese sample was marginally lower. These variations can be attributed to several interacting factors. Tic disorders are known for their characteristic waxing and waning, with symptom severity and frequency shifting [21], often due to environmental factors (e.g anxiety, stress) [16]. Previous researchs suggested that Chinese children encounter greater academic and related stressors in comparison to American children [22,23], a factor that could potentially engender greater variability in tic severity over time and, subsequently, impact the stability of measurements such as the PTQ. Further complicating the reliability of such assessments are external variables, including pharmacological interventions or behavioral therapies like CBIT, which can precipitate changes in symptomatology. Whereas Chang et al. [6] and Ricketts et al. [8] delineated study inclusion criteria that necessitated participants to be either unmedicated or on a consistent medication regimen for a period of 4–6 weeks prior to the commencement of the study, the present investigation did not stipulate such requirements, potentially introducing additional variability.

### Validity of the PTQ

The concurrent validity of the PTQ was supported by strong correlations with the YGTSS. These findings align with Chang et al. [6] and Ricketts et al. [8], affirming the validity of the PTQ as a measure of tic severity. The slight variability of concurrent validity across studies might be attributed to cultural perceptions and evolving mental health awareness as described previously. The psychometric validation of the YGTSS has been extensively conducted within Western contexts [4,18,24,25]. This focus raises concerns about its applicability to the Chinese population, whose cultural, linguistic, and socio-behavioral subtleties might not be fully captured by the YGTSS’s metrics. Such discrepancies suggest that the YGTSS’s comprehensive evaluations might not align with the nuanced Chinese perspectives on tic severity and its implications [26]. Furthermore, the dynamic between clinician-administered YGTSS interviews and parent observations on the PTQ could introduce subtle differences in tic severity ratings. Collectively, these studies underscore the PTQ’s validity as a time-efficient alternative to clinician-administered assessments.

The findings in this study also provide evidence for the discriminant validity of the PTQ. Results suggest that the PTQ is measuring tics distinctly from symptoms of other comorbid conditions, such as OCD (as indicated by the CY-BOCS score) and various behavioral and psychological symptoms (as represented by specific subscales of the CPRS-48).

### Limitations and future directions

One notable limitation of our study is the relatively small sample size used for the test-retest reliability analysis (n = 31). Although this subset was randomly selected and is considered adequate for preliminary reliability testing, larger sample sizes are crucial for more robust and generalizable results. Furthermore, the inherent variability of tics, which can fluctuate in frequency and intensity due to factors such as stress, anxiety, and environmental changes [15,16], presents specific challenges. While test-retest reliability is a commonly used method to assess the stability of instruments over time, it may not fully capture the dynamic nature of tic disorders. Nevertheless, this method provides valuable insights, particularly for assessing the consistency of more stable symptoms.

To overcome the limitations of test-retest reliability, future research should explore alternative methods such as inter-rater reliability, where multiple observers assess the same participants, and longitudinal studies that monitor tic severity over extended periods. These approaches would provide a more comprehensive understanding of the reliability and validity of tic assessment instruments. Additionally, considering the effects of medication on tic severity is crucial, as our study did not require participants to be unmedicated or maintain a stable dosage for 4–6 weeks prior to the study, which could have impacted the reliability of our findings.

Further research should also investigate the congruence between mothers’ and fathers’ scores on the PTQ to gain insights into the consistency of parental reporting and to explore potential gender differences in symptom perception and the influence of socio-cultural norms. Moreover, the lack of psychometric instruments specifically adapted and validated for the Chinese context, such as the YGTSS and the CY-BOCS, directly impacts the validation process of the PTQ. There is a critical need for a concerted effort to develop a broader range of tic severity questionnaires in China to ensure cultural appropriateness and effectiveness.

## Conclusion

In conclusion, this study showed that the PTQ demonstrated acceptable reliability and good validity in a sample of Chinese children with tic disorders. Overall, findings provide support for the PTQ’s standing as a psychometrically-sound instrument for assessing tic severity in children with tic disorders within Chinese clinical and research settings. Moreover, the analysis of item difficulty underscores the importance of further cultural adaptation of the PTQ. Tailoring the PTQ to better fit the cultural context will enhance its effectiveness and accuracy in diagnosing and managing tic disorders across diverse patient populations.

## Supporting information

S1 File (ZIP)
